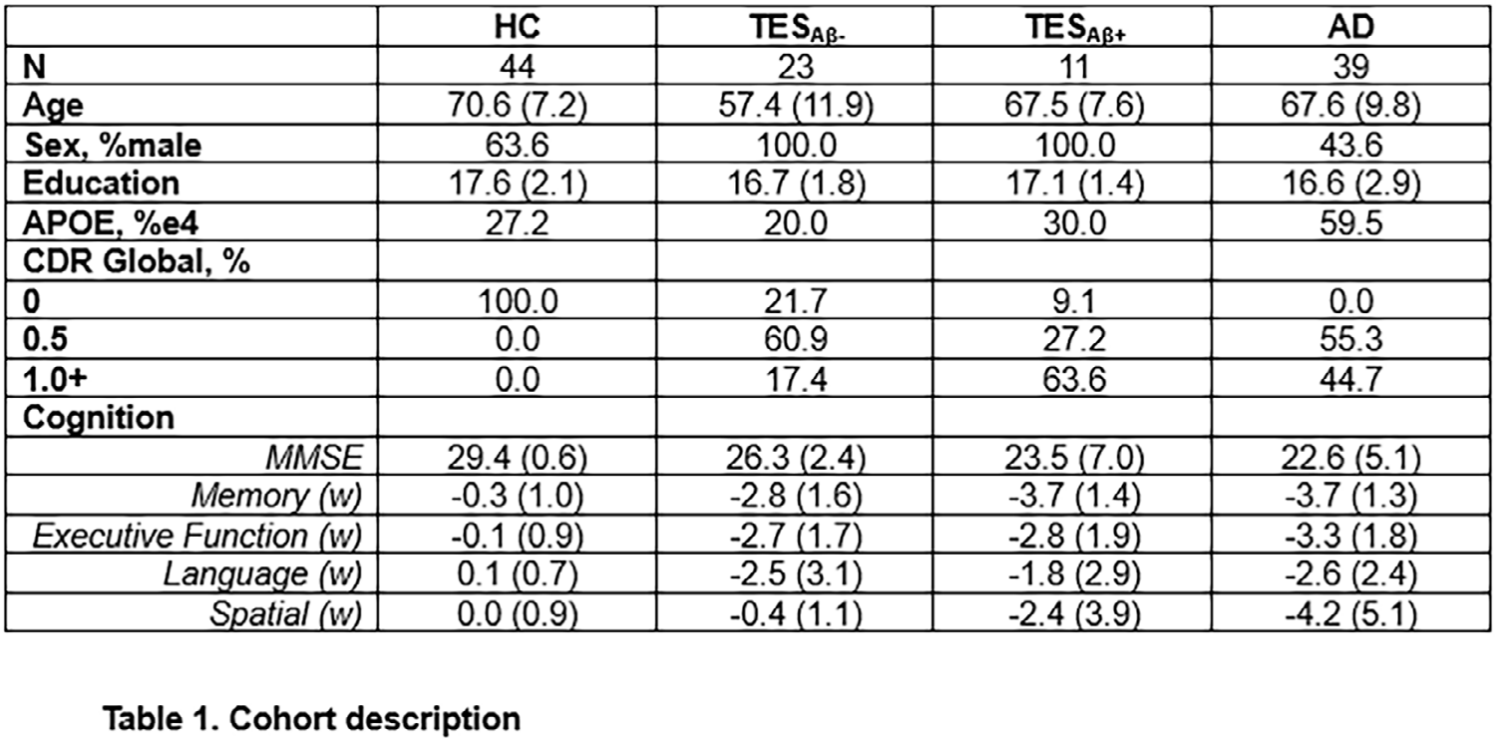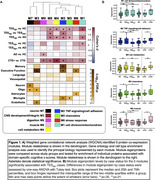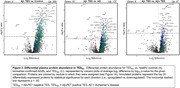# Large‐Scale Analysis of the Plasma Proteome and Cognitive Correlates in Traumatic Encephalopathy Syndrome and Chronic Traumatic Encephalopathy

**DOI:** 10.1002/alz.092953

**Published:** 2025-01-09

**Authors:** Rowan Saloner, Kaitlin B. Casaletto, Lawren VandeVrede, Jose F. F Abisambra, Glenn E. Smith, Salvatore Spina, Lea T. Grinberg, William W. Seeley, Bruce L. Miller, Joel H. Kramer, Gil D. Rabinovici, Breton M. Asken

**Affiliations:** ^1^ Memory and Aging Center, UCSF Weill Institute for Neurosciences, University of California, San Francisco, San Francisco, CA USA; ^2^ UCSF Alzheimer's Disease Research Center, San Francisco, CA USA; ^3^ Memory and Aging Center, UCSF Weill Institute for Neurosciences, University of California San Francisco, San Francisco, CA USA; ^4^ University of Florida, Gainesville, FL USA; ^5^ 1Florida Alzheimer's Disease Research Center, Gainesville, FL USA; ^6^ Memory & Aging Center, Department of Neurology, University of California in San Francisco, San Francisco, CA USA; ^7^ Memory and Aging Center, Weill Institute for Neurosciences, University of California, San Francisco (UCSF), San Francisco, CA USA; ^8^ Weill Institute for Neurosciences, University of California, San Francisco, San Francisco, CA USA; ^9^ Fixel Institute for Neurological Diseases, Gainesville, FL USA

## Abstract

**Background:**

Chronic traumatic encephalopathy (CTE) is a neurodegenerative tauopathy specific to individuals with repetitive head trauma. Traumatic encephalopathy syndrome (TES) is the proposed clinical syndrome resulting from CTE with or without other contributing neuropathologies. Pathophysiological mechanisms driving CTE and underlying TES symptoms are not understood. Using network‐based bioinformatics, we conducted the first large‐scale analysis of the plasma proteome in TES and CTE.

**Methods:**

We leveraged a proximity extension assay (Olink Explore) to quantify 2,778 plasma proteins in 34 TES, 39 biomarker‐confirmed Alzheimer’s disease (AD), and 44 controls (HC; Table 1). TES was subdivided on Aβ‐PET status (23 TES_Aβ‐_, 11 TES_Aβ+_). Secondary analyses focused on antemortem plasma ofrom brain donors with autopsy‐confirmed CTE (N=8) and without (N=12). Differential abundance and network analyses of proteomic signatures were annotated via gene ontology and cell type enrichment. Protein co‐expression modules were age‐ and sex‐adjusted, and compared between groups using analysis of variance (Tukey’s post‐hoc with Cohen’s d). Within TES_Aβ‐_, differential Spearman’s correlations examined individual protein associations with cognitive composites (memory, executive function, language, visuospatial) and co‐expression modules were tested for overrepresentation of cognitive‐associated proteins using Fisher’s exact tests with false discovery rate (FDR) correction.

**Results:**

Differential abundance analysis revealed a bias towards increased plasma protein abundance in TES_Aβ‐_, with enrichment for mitochondrial proteins (e.g., MECR, TOMM20). Network analysis identified 9 protein co‐expression modules (M1‐M9), of which 5 were elevated in TES_Aβ‐_, but not TES_Aβ+_ or AD, compared to HC (M1‐cell division/mitochondrion, d=1.4; M2‐TNF signaling/cell adhesion, d=0.72; M4‐cell metabolism, d=0.90; M5‐chemokine, d=0.80; M8‐muscle, d=0.88; Figure 1). Metabolic and immune‐linked signals in TES_Aβ‐_ were replicated when comparing autopsy‐confirmed CTE to plasma of brain donors without CTE or those living with AD and HC. Within TES_Aβ‐_, M2‐TNF signaling was highly enriched for proteins negatively associated with memory (FDR‐p=8.5e‐08) and executive function (FDR‐p=2.3e‐6).

**Conclusions:**

Network‐based proteomics identified elevated peripheral molecular signatures that differentiated TES and autopsy‐confirmed CTE from healthy older adults and biomarker‐confirmed AD. Mitochondrial and inflammatory pathway dysregulation may be uniquely upregulated in CTE and underly cognitive changes in TES. Future protein‐specific assessment (Figure 2) will inform biomarker and therapeutic targets in older adults at greatest risk for CTE.